# Eating Alone or Together among Community-Living Older People—A Scoping Review

**DOI:** 10.3390/ijerph18073495

**Published:** 2021-03-27

**Authors:** Amanda Björnwall, Ylva Mattsson Sydner, Afsaneh Koochek, Nicklas Neuman

**Affiliations:** Department of Food studies, Nutrition and Dietetics, Uppsala University, Box 560, 75122 Uppsala, Sweden; ylva.mattsson.sydner@ikv.uu.se (Y.M.S.); afsaneh.koochek@ikv.uu.se (A.K.); nicklas.neuman@ikv.uu.se (N.N.)

**Keywords:** commensality, eating alone, older people, loneliness, food intake

## Abstract

Research on healthy aging commonly concerns problems related to loneliness and food intake. These are not independent aspects of health since eating, beyond its biological necessity, is a central part of social life. This scoping review aimed to map scientific articles on eating alone or together among community-living older people, and to identify relevant research gaps. Four databases were searched, 989 articles were identified and 98 fulfilled the inclusion criteria. In the first theme, eating alone or together are treated as central topics of interest, isolated from adjoining, broader concepts such as social participation. In the second, eating alone or together are one aspect of the findings, e.g., one of several risk factors for malnutrition. Findings confirm the significance of commensality in older peoples’ life. We recommend future research designs allowing identification of causal relationships, using refined ways of measuring meals alone or together, and qualitative methods adding complexity.

## 1. Introduction

Research on health and aging commonly concerns problems related to loneliness [1] and food intake [2]. The prevalence of perceived loneliness and food-related problems increases with age and often has a multifactorial origin, due to common age-related physiological, socio-economic, and psychological changes [2,3]. Furthermore, loneliness and food-related problems are not independent aspects of health since eating, beyond its biological necessity, is a central part of social life [4]. In fact, the practice of sharing a meal—commensality—has been identified as one key social influence on eating behavior in later life, which can stimulate greater pleasure from food and may improve nutritional status [5].

The number of older people is growing [6] and so is the requirement to stay in community-living and independently for long periods of time. Nevertheless, reviews on food in later life that illuminate the role of eating alone or with others have primarily focused on institutionalized people [7,8], thus overlooking a large community-living population. Consequently, the aim of this article was to map the currently available scientific articles on eating alone or together with others among community-living older people. The research question was: which topics, methods, and main results are dominating the literature? Additionally, we aimed to identify research gaps relevant for future studies. With this aim, of searching broadly across disciplines, methodological approaches, outcomes (sociocultural, psychological, dietary, etc.), and geographical locations, a scoping review was chosen as our method.

## 2. Materials and Methods

This scoping review followed the framework established by Arksey and O′Malley [9] and enhanced by Levac et al. [10].

### 2.1. Search Strategy

To find relevant studies a block search method was used, including three blocks: target group, meals and social aspects. In order for an article to be identified in the list of results, it was necessary to have a hit of one or more search terms in each of the three blocks. Databases and search terms were established by first consulting with a librarian who was experienced in literature searches in public health sciences, and then by carrying out several test runs to calibrate the search. Databases used were PubMed, Web of Science, Cinahl, and PsycInfo (search terms in Appendix A). To enable a broad search, no limits were set regarding the time of publication (see full electronic search strategy in Appendix B). The literature search was conducted in December 2018 and updated in June 2020. The list of results was managed in a bibliographic software (EndNote X8; Clarivate, Philadelphia, PA, USA); after manual removal of duplicates (by software and manually), the total number of unique hits were 989.

### 2.2. Study Selection Process

**Figure 1:**
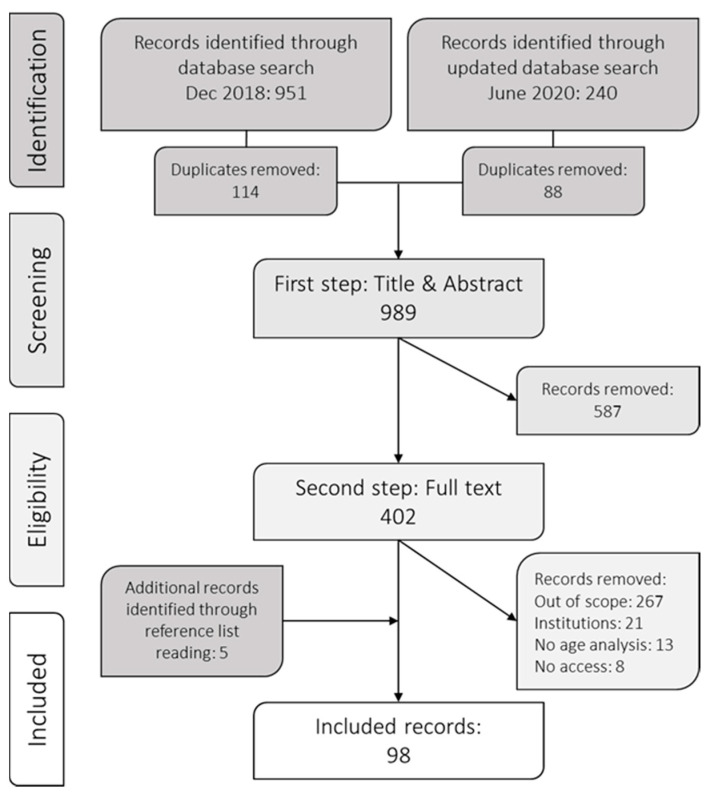
Flowchart of screening process.

Title and abstract for all the hits were screened by two independent readers and were either included for further review or excluded. Inclusion criteria for the first step of screening were: English or Swedish language; human studies; no study protocols; older people as target group, or a part of target group (e.g., 18 and above) and including social aspects of the meal. The screening process is summarized in Figure 1. During the first step, all abstracts indicating that social aspects of meals could have potentially been studied were included. Examples of such terms were “social participation,” “social support,” and “social isolation.” Moreover, “older” means different things in the literature. We therefore followed the definitions used by the respective authors. In other words, if the author(s) of an article wrote that the study included older people, or if the age range of a study was wide with no explicit exclusion of older people, it was included in this review. Disagreements and uncertainties were resolved after deliberation by A.B. and N.N.

In the second step, full texts of the articles were collected and read. The terms indicating social aspects were scanned and all studies not including eating alone or eating together with others were excluded. To reduce the risk of studies being included more than once in the results, review articles were excluded. Only the articles including community-living older adults were included for further review compared to living institutionalized (e.g., long-term care, hospital, etc.). “Community-living” was defined as living independently, and studies were included when this was made explicit or unless another living situation was described. Several articles lacked analyses or discussions related to age, even though older people were included in the study sample. These articles were therefore excluded, since the lack of any detail about older people meant that it did not reveal any information corresponding to the aim. Lastly, as in the first step, disagreements and uncertainties were resolved after deliberation by A.B. and N.N.

### 2.3. Data Analysis

When charting the data, three main categories were created to get an overview of the results that were most central to our research question. The first category included articles where eating alone or together was the central topic, meaning that it was clear from the build-up of the articles, the methodology sections, the results, and the discussions that the role of eating alone or not was at the core of the research. In the second category, eating alone or together was included as one aspect in a larger context, where eating alone was discussed but not dominant in the results or discussions. In other words, these aspects were formulated as relevant for the research, but not at the core. The third category included articles where eating alone or together was peripheral although it occurred in the method or as a minor part of the results.

In the next section, we present the findings from the first and second categories. Since articles in the third category did not contribute substantially to the aim, they are presented in Appendix C and Table A3. Our findings are not synthesized or weighed together [9,10]. Nonetheless, the large body of material included are collated and an overview is presented with relevant examples highlighted.

## 3. Results

### 3.1. Study Characteristics

After the screening, 98 articles were included. The majority used quantitative methods and consist of cross-sectional surveys (*n* = 45), longitudinal surveys (*n* = 9), and experimental studies (*n* = 4). About one-third used qualitative methods, most commonly individual interviews (*n* = 17), focus group interviews (*n* = 7), other study designs or a combination of qualitative study designs (*n* = 7). Nine articles, combining quantitative and qualitative methodologies were included. Most articles came from Northern America (40%), Northern Europe (24%), and Eastern Asia (16%). The most prevalent countries among all studies were USA (*n* = 33), UK (*n* = 14) and Japan (*n* = 9), followed by a wide spread of countries contributing with five or less (Appendix D).

### 3.2. Eating Alone as Central Topic

Eating alone is the central topic in one-third of the articles (*n* = 30), consisting of both quantitative and qualitative studies with several different aims and methods. Prominent topics examined were: dietary behavior, depressive symptoms, household eating arrangements, loneliness, and coping with grief. The most relevant examples for our study aim are described below, and all articles included are presented in Table 1.

#### 3.2.1. Quantitative Studies

In most quantitative studies, different health variables in relation to eating alone were surveyed, such as dietary behavior [11,12], subjective well-being [13], metabolic syndrome [14], or mortality [15,16]. One example was a longitudinal study (*n* = 2584), testing the combined effect of nutritional status and eating alone, a variable based on the question “Are there others present during your meals?” on cognitive changes [17]. Nutritional status was found as one salient predictor of cognitive decline, and women who had a compromised nutritional status at baseline and ate alone had a greater cognitive decline than those eating together. The authors conclude that nutritional programs for older people should focus on what they eat as well as with whom. A study using a cross-sectional design (*n* = 2196), examined subjective well-being among older people living alone [13]. Those who ate together less than once a month had lower rates of subjective health, food diversity, and food intake frequency than those who ate together more often. Another study, with a similar topic and design (*n* = 83,364) examined dietary behavior and body weight status in relation to eating alone and found that men who exclusively ate alone were more likely to skip meals than men who ate with others [11]. The authors concluded that among men, eating and living alone may be jointly associated with a higher prevalence of both obesity and underweight, as well as unhealthy food habits.

Eating alone was examined in relation to depressive symptoms and, compared to eating together, it was associated with higher rates [18,19,20,21,22]. One of the studies had a longitudinal design (*n* = 37,130); apart from a generally increased risk of depressive symptoms, the authors argued that among men, the negative effects ascribed to eating alone could be amplified when living alone [20]. Eating together with company was studied by asking “Who do you usually have meals with?” with the response alternatives: “no one”/“spouse”/“children”/“grandchildren”/“friends”/“other.” Furthermore, in three of the articles, it was suggested that those who ate alone, yet lived with their families, seemed to be at a particular risk [19,21,22]. This category, also used in Torres et al. [23], was only represented by small proportions of the samples (around 5%).

Another set of studies looked at eating arrangements, without any outcomes on health and well-being. Yates and Warde [24] used an online survey to look at eating alone and living arrangements among adults in British households (*n* = 2784). They found that older respondents were more likely than younger to eat with companions if they lived with others, whereas the oldest respondents living alone were least likely to eat with others. Jacobson et al. [25] used questionnaires (*n* = 625) in combination with qualitative interviews (*n* = 9) in the Cyprus context to examine households′ food expenditure as well as “food as a shared meal in the sociological sense” (p. 677). They found that there was a relatively high expenditure on food for home consumption by older couples, which may be explained by older people providing food when younger family members (i.e., children and grandchildren) frequently come to their homes for shared meals. Furthermore, a Japanese study examining the frequencies of family commensality, comparing differences between people under and over 60 (*n* = 242), found that those older than 60 had the highest commensal frequency; moreover, it was the only age group exceeding the national average rate of commensal meals [26].

Lastly, three experimental studies were included in this category. One randomized controlled pilot study examined the effects of a mealtime intervention in older participants (*n* = 50 control group; *n* = 50 intervention group) living alone and in self-reported risk of social isolation [27]. During the intervention the participants met a volunteer to cook and share a meal together, once a week for eight weeks. Improvements were evident within the treatment group, but also relative to participants in the control group regarding food enjoyment. The authors concluded that there was a gap in the current services offered to older adults and suggested that a combination of social and nutritional support would be of importance for older people living alone. However, they were also conscious about the study being underpowered due to the low sample size.

The other two experimental studies examined the social facilitation of eating. Oral nutritional supplements were tested in the first study [28], where the participants (*n* = 21) were their own controls, attending one individual session and one group session in which participants brought two friends. Eating together resulted in a 60 percent increase in the total energy intake, compared to eating alone. The other study tested whether the social facilitation of eating occurred even in the absence of other people, when eating in front of a mirror (*n* = 16) or a picture of oneself (*n* = 12) [29]. The participants ate more and rated that the snacks had better taste when eating in front of a mirror or a picture than when eating in front of a wall. However, these studies also have low sample sizes.

#### 3.2.2. Qualitative Studies

When examining different aspects of older peoples′ lives or situations, eating alone or together with others became a central topic in several qualitative studies. Eating alone was mainly described as a negative experience by participants in the interview studies [30,31,32]. Older people of low weight described eating alone, social isolation, and stressors as the main influences on their eating patterns [30]. Respondents who experienced social isolation and were living with others, yet eating alone, also expressed feelings of being a burden. In an article on the role of the meal for retired women, both cohabiting and single-living, the meal was expressed as a gift [31]. However, for the widowers, the meaning of cooking and eating was expressed as lost, and the authors warned that this group was at nutritional risk. In an article focusing specifically on widowers, Vesnaver et al. [32] explored loss of commensality and found that eating alone led to fewer regular meals and less time spent on food. However, participants also said that even if mealtimes were negatively affected by being alone at the table, cooking and eating could still be pleasurable [32].

In contrast to these studies, meals eaten alone at home was described as being enjoyable by single-living older people, in a qualitative study on domestic and communal meals [33]. For example, dining in alone was linked with feelings of contentment and peacefulness. Additionally, freshness and variety of foods were considered of higher priority than commensality. The author writes:

“Therefore, whilst dining in was, for the main part, experienced alone it was not described by participants as a lonely event. By contrast, dining in alone was perceived in practical terms and, at times, symbolic of independence, competence and control” [33].

(p. 38)

A study by Keller et al. [34] explored the meaning and experiences of mealtimes, and the article illuminates perspectives of persons with dementia and their primary family partners in care. Participants described strategies used to support social engagement during mealtimes, such as making the meals an important ritual and not a task, using conversation aids during meals, or eating together in a calm environment without distractions. Even though it could be more challenging as the dementia progressed, families enjoyed socializing by eating out, or getting together with other social groups.

One article, which stood out in its methodology, presented a formative evaluation study of mixed-reality solitary meals in virtual environments among older adults with mobility impairments [35]. The participants had a meal alone, using a head-mounted display showing two different virtual environments: a kitchen and a park. The interviews revealed that the preferred eating environments were dependent on which meals the participants were served, but the kitchen, where they usually ate alone, was reported as the perfect eating-alone environment. 

**Table 1:** Articles in the first category: central topic.

Reference	Study Design	Participants	Aim	Results
	QUANTITATIVE			
	*Cross-sectional*			
Davis, M. A., et al., 1988. **USA** (Brief report) [36]	Cross-sectional study	*n* = 4.402, 55–98 years	To examine associations between living arrangement and several eating behaviors.	Those living alone consumed more meals alone, ate higher proportion of food away from home and skipped more meals, than those living with a spouse.
Ishikawa, M., et al., 2017. **Japan** [13]	Cross-sectional study	*n* = 2.196, 65–90 years	To examine the relationships between eating together and subjective health, frailty, food behaviors, food accessibility, food production, meal preparation, alcohol intake, socioeconomic factors and geography among older Japanese people who live alone.	Those who ate together less than once a month (47% men, 23% women) had significantly lower rate of subjective health, food diversity and food intake frequency than those who ate together more often. The factors most strongly related to eating together less than once a month were not having food shopping assistance, not receiving food from relatives or neighbors, income, daily alcohol intake and frailty (for men only).
Kimura, Y., et al., 2012. **Japan** [18]	Cross-sectional study	*n* = 856, 65+	To clarify the relations between eating alone and geriatric functions such as depression, quantitative subjective quality of life (QOL), activities of daily living (ADL) and dietary status of community-living Japanese older people.	Those who usually ate alone (*n* = 248, 33.2%) were significantly more depressed and had lower QOL-score, compared to those who usually ate with others. Among the *n* = 697 subjects who lived with others, *n* = 136 (19.5%) ate alone.
Kuroda, A., et al., 2015. **Japan** [19]	Cross-sectional study	*n* = 1.856, 65–94 years	To examine the association between social engagement and depressive symptoms with a particular focus on eating alone and how the association changes along the aging and mental frailty trajectories.	14.6% were eating alone and 6% were eating alone despite living with family members. Eating alone was associated with higher risks of both mild and severe depression. Those who lived with their families yet ate alone were found to be at particular risk.
Kwon, A. R., et al., 2018. **South Korea** [14]	Cross-sectional study	7.725 adults, 19+, mean age 47.1	To investigate the association between eating alone and the metabolic syndrome (MetS) and to identify whether sociodemographic factors can modify this association.	There was a significant dose-response association between eating alone and MetS, independent of relevant confounders including sociodemographic and life style factors. Individuals who ate alone 2 or more times per day showed higher frequency of living alone, having no spouse, skip meals, and less eating out (*p* < 0.05). The association between eating alone and MetS was dependent on sex and presence of spouse.
Lee, S. A., et al., 2016. **South Korea** [21]	Cross-sectional study	*n* = 4.181, 20+	To investigate the association between the dinner companion and depression, and the differences in this association by sex, living arrangement and household composition.	Those who ate alone had higher depression rate compared to those who ate with family. The subgroup analysis indicated that men, those who live with others and those living in a second-generation household who ate alone had greater odds of having depressive symptoms.
Locher, J. L., et al., 2005. **USA** [12]	Cross-sectional study	*n* = 50, 60–95 years, mean age 77.1	To investigate the effect of the presence of others on caloric intake in homebound older adults.	40% of participants consumed all meals alone, 28% consumed all meals with someone else and 32% ate some meals with and some meals without others. Participants consumed more calories for all meals in the presence of others compared to eating alone. Meals in the presence of others indicates an average of 114 kilocalories more per meal. After controlling for others′ presence at meals, the presence of others in the household had no significant effect on caloric intake.
Motteli, S., et al., 2017. **USA** [37]	Cross-sectional study	Part I: *n* = 502 females, 19–95 years; Part II: *n* = 262, 19+	To investigate women’s regular eating networks and whether these were associated with dietary behavior and body weight.	Women shared their meals most frequently with family members. Those who dined more often with healthy eaters reported a higher diet quality and lower BMI, on average. Part II showed that different diet-related factors were correlated between women and their most important eating companions. Higher diet quality of the eating companions was associated with lower BMI in women.
Takeda, W. et al., 2018. **Japan** [26]	Cross-sectional study (administered in face-to-face interviews)	*n* = 242, 20–85 years (*n* = 63, 60+)	To examine frequencies of family commensality among Japanese adults in two metropolitan areas.	Family commensality was less frequent among those living alone, and with only non-partners including adult children, parents, and non-family members, than among those living with partners. Mean frequencies for family commensality were highest for those over 60, for all meals. Adults 60+ were the only group to exceed the national average rate, with rates being much lower among younger groups, those living with non-partners, and full-time workers.
Tani, Y., et al., 2015. **Japan** [11]	Cross-sectional study	*n* = 82.364, 65+	To examine whether eating alone is associated with dietary behaviors and body weight status, and assessed the modifying effects of cohabitation status in older Japanese adults.	16% of men and 28% of women sometimes or exclusively ate alone. Among those who exclusively ate alone, 56% of men and 68% of women lived alone. Depending on cohabitation status, eating alone and living alone may be jointly associated with higher prevalence of obesity, underweight and unhealthy eating behaviors in men.
Torres, C. C., et al., 1992. **USA** [23]	Cross-sectional study (administered in face-to-face interviews)	*n* = 424, 58+, mean age 71.9	To examine four identified living and eating arrangement groups and what social network, functional disability etc. determine such group membership.	Older people with greater number of companions and percentage of kin in social network, were less likely to both live and eat alone. Men with higher income and age were also less likely to live and eat alone. Most older adults either live and eat alone or live and eat with others, mixed living/eating arrangements are rare.
Wang, X., et al., 2016. **China** [22]	Cross-sectional study (administered in face-to-face interviews)	*n* = 7.968, 60+	To explore the relationship between eating alone and geriatric depressive symptom.	17% of the participants ate alone and 9% had depressive symptom, those who ate alone but lived with others had a significant increased odds of depressive symptoms.
Yates, L. & Warde, A. 2017. **UK** [24]	Cross-sectional study	*n* = 2.784, 18+	To examine meal arrangements in British households in 2012.	Eating alone was associated with simpler, quicker meals, and most commonly took place in the morning and midday. Those living alone eat alone more often but at similar meal times, and they take longer over their lone meals.
	*Longitudinal*			
Huang, Y. C., et al., 2017. **Taiwan** [15]	Longitudinal study (10-year follow up)	*n* = 1.894, 65+, mean age 72.9	To investigate the sex-specific association between eating arrangements and risk of all-cause mortality among community-living older adults.	63% of men and 56% of women ate with others three times a day. Those who ate with others were more likely to have higher meat and vegetable intake and greater dietary quality than those who ate alone. Eating-with-others two or three times per day was an independent survival factor for older men, but not for women.
Li, C. L., et al., 2018. **Taiwan** [17]	Longitudinal study (4- and 8 year follow-ups)	*n* = 2.584 baseline, *n* = 2064 4-year follow-up, *n* = 1570 8-year follow-up. 65+, mean age 74	To test the combined effect of two hazards, the risk of malnutrition and eating meals alone, on the cognitive changes among a representative sample of older Taiwanese individuals over an 8-year period.	Nutritional status was a salient predictor for cognitive decline among participants. Female respondents with compromised nutritional status at baseline and eating meals alone exhibited greater decrease in mental-status scores compared with those who had a normal nutritional status and who were eating their meals with others.
Tani, Y., et al., 2018. **Japan** [16]	Longitudinal population-based study (3-year follow up)	*n* = 71.781, 65+	To examined the association between eating alone and mortality accounting for confounding factors among older Japanese adults.	Compared with men who ate and lived with others, the hazard ratio after adjusting for confounding factors was significantly higher for men who ate alone yet lived with others. Among women, there was no statistically significant association, neither for women who ate alone yet lived with others or for women who ate and lived alone.
Tani, Y., et al., 2015. **Japan** [20]	Longitudinal population-based study (3-year follow up)	*n* = 37.193, 65+	To examine the association between eating alone and depression in the context of cohabitation status in older adults in Japan.	After adjustment for confounding factors, depression onset in men who ate alone compared with those who ate with others was significantly higher for those living alone. Among men, the effect of eating alone on depression may be reinforced by living alone, but appears to be broadly comparable with women.
	*Experimental*			
McAlpine, S. J., et al., 2003. **UK** [28]	Experimental study	*n* = 21, 60–79 years	To examine whether nutritional supplements are less preferred and less likely to be selected than other energy-dense foods, and whether eating alone further reduces intake relative to eating in a social setting.	Favorite flavor of sip-feed (nutritional supplements) compared well with other more familiar foods and was selected as part of a snack. Intake increased by 60% when consumed in a group setting compared with eating alone.
McHugh Power, J. E., et al., 2016. **Ireland** (Pilot study) [27]	Experimental pilot study (randomized controlled trial design)	*n* = 100, 55+	To investigate the effects of a novel mealtime intervention (including 50 volunteers, 55+) on self-efficacy, food enjoyment and energy intake on older adults, living alone in self-reported risk of social isolation.	Participants in treatment showed improvements relative to those in control group at borderline significance (*p* = 0.054) for self-efficacy and at significance for food enjoyment. No clear effects for energy intake or social cognitive factors.
Nakata, R. & Kawai, N. 2017. **Japan** [29]	Experimental study	Experiment 1: *n* = 16, 65–74 years, mean age 68.4; Experiment 2: *n* = 12, 66–74, mean age 68.9	To analyze and answer whether the social facilitation of eating occur without the actual presence of other individuals.	Older and younger participants ate more popcorn and rated them better tasting in the self-reflecting condition than in the monitor condition. Furthermore, a similar observation of “social” facilitation of eating was made when participants ate popcorn in front of a static picture of themselves eating, suggesting that static visual information of someone eating food is enough to produce the “social” facilitation of eating.
	QUALITATIVE			
Boyer, K., et al., 2016. **Australia** (Brief report) [38]	Focus group interviews and questionnaire	*n* = 41 older adults	To explore an innovative social eating program model for older Tasmanians, from the perspectives of its participants.	The program was meeting the social eating needs of its participants and nurturing a sense of community.
Keller, H. H., et al., 2015. **Canada** [34]	Interviews (once a year in 3 years, individual or duo)	*n* = 27 families, one older person with dementia and at least one family care partner	To explore the meaning and experience of mealtimes for families living with dementia.	Strategies to support quality mealtimes were devised by families: living in the moment, maintaining social engagement and continuity of mealtime activities.
Korsgaard, D., et al., 2019. **Denmark** [35]	Formative evaluation study	*n* = 7, 74–86 years	To address the question: What virtual environment do mobility-restricted older Danish adults perceive as engaging and suitable for pleasurable, mixed-reality solitary meals?	When evaluating a mixed-reality eating prototype, safety, realism, practicality, social acceptability, time, palatability, and indoor-outdoor considerations are found to be important aspects of food environment.
Martin, C. T., et al., 2005. **USA** [30]	Individual interviews	*n* = 8 women, 65+	To investigate the factors that influence the dietary practices and eating patterns of low-weight (BMI <24) older adults and to examine the nutritional advice given by healthcare providers.	Eating alone, social isolation, and stressors are the main reasons for low weight, reported by participants.
McHugh, J., et al., 2015. **Ireland** (Letter to the Editor) [39]	Individual and focus group interviews	*n* = 6 older adults and *n* = 10 healthcare professionals	To investigate the significance of mealtimes for older adults living independently in the community, as well as the opinions of healthcare professionals working with this population.	Older adults were unaware of relationships between nutrition and health, but saw importance of sharing mealtimes with others. Healthcare professionals were more likely to discuss nutritional needs.
Saeed, A., et al., 2019. **UK** [40]	Individual and focus group interviews	*n* = 42, 59–89 years	To examine psychosocial barriers and facilitators to attending community-based social eating opportunities for older adults.	Four themes were identified that related to the importance of offering more than food (combine with other activity or to meet new friends); participants’ social identity (being with my kind of people and labelling of groups); taking the first step (going together and having personal connection; and embarrassment and self-consciousness about physical health.
Sidenvall, B., et al., 2000. **Sweden** [31]	Individual interviews	*n* = 63 women, 63+	To delineate the meaning of preparing, cooking, and serving meals among retired single living and cohabiting women.	The meal could be seen as a gift, cohabiting women were cooking with duty and joy. For widows the whole meaning of cooking and eating was described as lost.
Thomas, N. & Emond, R., 2017. **UK** [33]	Individual interviews and 5-day food diary	*n* = 10, 60–88 years	To explore the perceptions and preferences of ten older people towards domestic and communal meals.	A number of key themes identified, including the meaning of mealtimes. Participants ate majority of meals at home alone. Despite this, dining alone was not necessarily experienced as lonely.
Vesnaver, E., et al., 2016. **Canada** [32]	Individual interviews	*n* = 15 women, 71–86 years	To explore loss of commensality among older widowed women in relation to food behavior.	Participants attributed changes to their food behaviors to the loss of commensality, including food choice, fewer regular meals, and reduced work of meal preparation.
	MIXED DESIGN			
Jacobson, D. S., et al., 2015 **Cyprus** [25]	Cross-sectional survey and individual interviews	*n* = 625 households (quant. survey), *n* = 9 households (qual. interviews)	To show the relationship between food as a shared good in the economic sense, and food as a shared meal in the sociological sense.	There was relatively high expenditure on food for home consumption by older couples, which may be explained by that older people provide with food when their younger family members (i.e., children and grandchildren), frequently come to their homes for shared meals.

### 3.3. Eating Alone as One Aspect

This category has the largest number of articles (*n* = 50) and covers a wide range of topics and aims, where eating alone or together with others constitute one aspect of the study, for example, in broader discussions about food habits, social isolation, widowhood, loneliness or the social context of eating. The most relevant examples for our study aim are described below, and all articles included are presented in Table 2.

#### 3.3.1. Quantitative Studies

The majority of articles in this category are based on quantitative studies, and half of these studies examine food-related problems in different ways. Whether eating alone was associated with lower food intake or higher nutritional risk varied in the different studies. For example, two studies used a questionnaire where eating alone, measured as “Do you eat one or more meals a day with someone?” was included and categorized as one risk factor for food intake [41,42]. They found that participants at high nutritional risk more often ate alone compared with those of lower nutritional risk. Similarly, participants categorized as being at nutritional risk (18%) were more likely to report eating alone (among other risk factors), as found in a study using a nutritional risk screening checklist on members of a congregate meal program (*n* = 8892) [43]. However, Posner et al. [44] did not find a significant association between eating alone and nutritional risk when they developed and tested the same checklist (*n* = 749). The checklist included the statement “I eat alone most of the time,” with the possible answers “Yes/No.” The last example is a study looking at dietary intake and eating patterns among older people in Israel [45]. The study found eating alone to be associated with lower food intake in men (*n* = 172) but not in women (*n* = 205), when the number of meals alone per week was included as a continuous variable.

Questionnaires including eating meals alone or together were also used in studies on social isolation and loneliness in relation to food-related problems [46,47]. One found that social isolation and loneliness were independently associated with higher nutritional risk, among 1200 randomly selected older people [46]. However, no association was found between frequency of sharing meals (“Do you share meals with others?” with response alternatives “most of the time”/“about half the time”/“infrequently”/“never”) and nutritional risk. Ferry et al. [47] conducted a mixed method study on a similar topic with older people, who lived alone and reported no more than five “emotionally meaningful” contacts per month (*n* = 150). One-third (32%) never shared a meal with family or friends and two-fifths (42%) reported inadequate food intake to cover nutritional needs. In both articles, the authors express concerns about loneliness and social isolation among older people and its effects on nutritional status.

The following four articles are presented to illustrate the variety of methods and topics touched upon in this category. The first example is an article that explored the benefits of active social engagement by evaluating different kinds of relationships [48]. The 133 participants were 60 years or older and generated 1506 social relationships in which interactions occurred at least once a month, of which 35 percent included regularly shared meals together. Social relationships that involved co-engagement in social and daily activities, such as shared meals, conveyed more social support, companionship, and positive social influence compared to the relationships that did not.

The second example is presented because it is the only included article with a single-blinded cluster controlled study design. The study aimed to understand whether older adults’ involvement in their own meals, as part of a rehabilitation program, could improve the health-related quality of life, muscle strength, and nutritional status [49]. From baseline to follow-up, a significant improvement was found in the intervention group compared to the control group in their health-related quality of life.

The third is a prospective population-based survey conducted in Botswana, with “eating meals alone” used as an indicator of diminished social support [50]. The study aimed to assess diminished function and lack of social support as indicators of short-term mortality (*n* = 372). Respondents who reported that they “usually eat meals alone” were assumed to have diminished social support, and this was found to be one of several significant discriminators for survival.

#### 3.3.2. Qualitative Studies

In the qualitative studies in this category, eating with others or alone seems to have been an inductive finding, meaning that nowhere is it stated in the method description (or in supplementary files, when such were available) that this was part of the original design. The majority aimed to examine a food-related topic such as food habits or food practices, although two studies considered mealtimes alone or with others when studying widowhood [51] and loneliness [52].

In two articles, the meal was described as an opportunity to meet other people and became a coping strategy toward loneliness [52,53]. Eating together represented one aspect of “social engagement,” which was identified as impacting diet quality among participants in a focus group study. The authors suggest that eating out with friends or family may be a key social activity [53]. Similarly, in a study examining loneliness in later life, food and beverage rituals were identified as aspects that maintain interactions with family and friends [52].

Being alone at the table was described as lonely and leading to having less motivation for cooking and eating in several studies [51,54,55,56]. The participant groups and topics of interest differed, such as food choice in later life among British households [54], food practices among Lesbian, Gay, Bisexual, and Transgender seniors [55] and newly bereaved older people coping with grief [51,56]. While all of the articles acknowledge the importance of the social context of food practices and meals to enhance nutritional status and well-being, the need to address dietary issues for grieving older adults was emphasized in particular in an interview study by Hegge [51] and a mixed method study by Johnson [56].

Loneliness in relation to mealtimes also emerged as a problem in two studies focusing on self-management of type 2 diabetes [57,58]. Not being able to eat the same food as the rest of the family and therefore eating separately was expressed as a problem, creating social isolation among participants in both studies. Another study, about life with type 2 diabetes, explored different views regarding a healthy diet among ethnic minorities in the Netherlands and found that all their participants were eating with their families [59]. Here, shared eating was described both as a support and a hindrance for changes in lifestyles and eating habits.

Overall, within this category qualitative findings tend to diverge in two different directions. In one, the meal is described as an opportunity to meet other people. In the other, lonely meals are said to result in less time spent on food practices and less regular meals. In one study with British older adults, the authors found both perspectives described by different participants relating to food practices and identity maintenance [60]. The participants were categorized into groups, based on their findings—“food lovers” and “nonfoodies”—and stated that “‘[b]eing alone at the table’ was the greatest threat to food activities and identity, especially for the food lovers who gained pleasure from the social aspect of their food activities” [60] (p. 5). The food lovers coped with being alone by cooking for friends and family, while the nonfoodies had different experiences, describing eating alone as difficult.

**Table 2:** Articles in the second category: one aspect.

Reference	Study Design	Participants	Aim	Result
	QUANTITATIVE			
	*Cross-sectional*			
Alberti Fidanza, A., 1984. **Italy** [61]	Cross-sectional study	*n* = 207, 65+	To identify nutritional knowledge, food preferences and life styles connected with nutritional process.	Participants demonstrates low levels of nutritional knowledge. Women and men show similar percentages of energy expenditure, both in time and in frequency in relation to sleep and sedentary activities. Participants are well integrated in family life and eat most meals with their families.
Ashida, S., et al., 2019. **USA** [48]	Cross-sectional study (administered in face-to-face interviews)	*n* = 133, 60+, mean age 75.4	To investigate whether social network functions (i.e., social support, companionship, social influence) are more likely to occur in relationships that involve active social interactions through co-engagement in activities compared to relationships that do not.	1506 social relationships in which interactions occurred at least once a month were analyzed, 52% involved engagement in social activities together and 35% involved eating together regularly. Social relationships that involve co-engagement in social and daily activities, such as eating meals together, conveyed more social benefits compared to the relationships in which they did not.
Boulos, C., et al., 2017. **Lebanon** [46]	Cross-sectional study	*n* = 1.020, 65+, mean age 74.9	To evaluate the association between three components of social isolation: social network, feeling of loneliness, commensality and nutritional status.	Social isolation and loneliness are independent risk factors for malnutrition. No significant association between the frequency of sharing meals and the risk of malnutrition. However people sharing most of the time of their meals with others were significantly less often malnourished.
de Castro, J. M., 1993. **USA** [62]	7- day food diary	*n* = 307, 20+ (*n* = 44, 65+)	To investigate age-related changes in food intake (participants were divided into four age groups).	The lower intakes that occur with age is a consequence of smaller meals, eaten relatively slowly. Older people (65+) were as responsive to a number of influences of intake as younger people e.g., time of day and number of people present.
de Castro, J. M., 2002. **USA** [63]	7- day food diary	*n* = 762, 20+ (*n* = 46, 65+)	To study age-related changes in the social, psychological, and temporal influences on food intake (participants were divided into four age groups).	Older people (65+) ate with fewer people present but were as responsive, as younger participants, to several factors e.g., social facilitation of intake and palatability, but showed blunted responses to self-reported hunger.
Dean, W. R., et al., 2014. **USA** [64]	Cross-sectional study	*n* = 2.785, 50+	To explore the relative associations of capital assets with food insecurity across socioeconomic class through a comparative analysis of the association of intimate social capital, individual evaluations of community social capital, government capital, and interactions between social and government capital across three socioeconomic stratifications.	Social capital was not uniformly associated with food-security status across the income stratifications. There was a significantly greater proportion of participants relying on gardening, hunting, fishing, and animal husbandry in rural than in urban counties. Rural residents ate meals with family and friends more than urban and regularity of meals with family and friends increased with income level.
Ferry, M., et al., 2005. **France** [47]	Cross-sectional study (administered in face-to-face interviews)	*n* = 150, 70-99 years, mean age 80.8	To determine the relationship between loneliness and nutritional status in persons aged over 70 years.	A large number of participants had an inadequate dietary intake and 21% had established undernutrition. 75% were widowed and 32% never shared a meal with family or friends.
Getty, M. D., et al., 2016. **USA** [65]	Cross-sectional study	*n* = 477, 59+	To assess the presence of these risk factors in limited-resource, community-living older adults (meal site participants) to inform the development of a nutrition education interventions.	More African Americans reported having a chronic illness or condition, eating alone, and sometimes not having enough money to buy food.
Holm, L., et al., 2016. **Denmark, Finland, Norway and Sweden** [66]	Analysis of two cross-sectional surveys	*n* = 4.808, 15+ (1997) and *n* = *n* = *n* = 8.248, 15–80 years (2012)	To compare data from 1997 and 2012, in Denmark, Finland, Norway and Sweden, regarding where, with whom, and for how long people ate, and whether parallel activities take place while eating.	Primary location for eating remained the home and the workplace, the practices of eating in haste, and while watching television increased. Propensity to eat alone increased slightly in Denmark and Norway, and decreased slightly in Sweden. Signs of individualization and in formalization could be detected.
Holmes, B. A. & Roberts, C. L., 2011. **UK** [67]	Cross-sectional study (administered in face-to-face interviews)	*n* = 662, 65+	To develop a single indicator of diet quality to provide a more accurate indicator of total diet in materially deprived men and women aged 65 and over, and to use this indicator to investigate risk factors associated with a poor quality diet in the low-income population.	The best quality diet was inversely associated with usually eating meals on one’s lap as opposed to at the table. For men, it was also inversely associated with difficulty chewing, whereas for women, it was inversely associated with current smoking and being 75 years or over. Results suggest that the social setting is an important determinant of diet quality in this group.
Holmes, B. A., et al., 2008. **UK** [68]	Cross-sectional study (administered in face-to-face interviews)	*n* = 234 men, 65+	To investigate the influence of those social, physical and other factors collected in the LIDNS on the food consumption and nutrient intake of men aged 65 years and over who participated in the survey.	Mean energy intakes fell below the estimated average requirement (84%), while mean intakes of several micronutrients fell below the reference nutrient intake. Results suggest that interventions need to focus on improving cooking skills, especially in men who live or eat alone.
Ishikawa, M., et al., 2018. **Japan** [69]	Cross-sectional study	*n* = 2.151, 65+	To clarify the food and health behavior factors associated with subjective well-being in older adults with a chronic disease living alone in the community.	Individuals with good subjective well-being had significantly higher rates than those with poor subjective well-being for satisfaction with meal quality and chewing ability, food diversity, food intake frequency, perception of shopping ease, having someone to help with food shopping, eating home-produced vegetables, preparing breakfast themselves, eating with other people, and high alcohol consumption.
Keller, H. H., et al., 2005. **USA** [70]	Cross-sectional study	Study 1: *n* = 193 (61 from geriatric clinics); Study 2: *n* = 149; Study 3: *n* = 97, 55+	Three studies testing the reliability and validity of an updated screening tool for nutritional risk (Seniors in the Community: Risk Evaluation for Eating and Nutrition II, SCREEN II).	Respective median scores on SCREEN II were 51, 49 and 52. Proportion responding “Yes” to “Do you eat one or more meals a day with someone?” was 33%, 42.3% and 55.7%, for Study 1, 2 and 3, respectively.
Nicholas, M., et al., 2020. **USA** [71]	Cross-sectional study	*n* = 25, 34+, mean age 59.9 (and *n* = 12 caregivers)	To examine everyday activities valued by people with aphasia (PWA) using the Life Interests and Values (LIV) Cards; to measure congruence between PWA and their caregivers on life participation goals.	PWA endorsed wanting to participate more in a wide range of activities, with common interests in walking/running, going to the beach and eating out, among others. PWA–caregiver activity agreement was fair to moderate with point-to-point agreement averaging 70%.
Porter, K., et al., 2016. **USA** [72]	Cross-sectional study	*n* = 289, 60+, mean age 74.6	To explore the associations between sexual orientation and the perceived social network and nutritional value of congregate meal programs (CMPs).	Sexual minorities were more likely to have non-kin-based social networks, reported higher levels of loneliness compared with heterosexuals and travelled seven times the distance to attend CMPs.
Posner, B. M., et al., 1993. **USA** [44]	Cross-sectional study	*n* = 749, 70+	To recommend items for a consumer awareness checklist for the American “Nutrition Screening Initiative” and to calibrate the instrument.	A revised 10 yes/no-item checklist was adopted and 24% of the Medicare population were estimated at high nutritional risk according to the checklist. No significant association was found between answering “Yes” to “I eat alone most of the time” and dietary inadequacy or perceived health.
Quigley, K. K., et al., 2008. **USA** (Research brief) [43]	Cross-sectional study	*n* = 8.892, 60+	To determine if there were differences by demographic variables in response rates to Nutrition Screening Initiative (NSI) among Oklahoma Older Americans Act Nutrition Program OAANP, congregate meal participants	50% of participants categorized at high nutritional risk reported “yes” to having an illness or condition that affected food eaten; eating alone; taking 3 or more medications; and inability to shop, cook and feed themselves.
Rosenbloom, C. A. & Whittington, F. J., 1993. **USA** [73]	Cross-sectional study (administered in face-to-face interviews)	*n* = 50 widowed and *n* = 50 married, 60+	To identify the effects of recent widowhood on nutritional behaviors.	Widowhood triggered disorganization and changes in the participant’s daily routines associated with food preparation and eating. 72% of the widowed reported loneliness at mealtimes since the death of their spouse and the widowed had a significantly lower Diet Quality score than the married (t = 8.74, *p* < 0.001).
Rugel, E. J. & Carpiano, R. M., 2015. **Canada** [74]	Cross-sectional study (administered in face-to-face interviews)	*n* = 14.221, 65+	To test hypotheses regarding direct/indirect pathways through which tangible and emotional/informational social support may facilitate adequate fruit and vegetable consumption.	Emotional/informational support was positively associated with adequate fruit and vegetable consumption. Neither social support form was directly or indirectly associated with adequate consumption in men. Adequate consumption was negatively associated with tangible support but positively associated with higher emotional/informational support in women.
Shahar, D., et al., 2003. **Israel** [45]	Cross-sectional study	*n* = 377, 60+ (*n* = 224, 65–74 years; *n* = 153, 75+)	To determine dietary intake and eating patterns of older persons in Israel and identify factors associated with low dietary intake.	Energy, fat, carbohydrates, vitamins E, C and B1 intake were significantly lower for people aged 75 and older. Low energy intake was associated with lower subjective health status for men (*p* < 0.01), poor appetite (*p* < 0.01) and more gastrointestinal problems (*p* < 0.05) for women and lower snack consumption (*p* < 0.01) for both sexes. Eating alone was significantly and independently associated with low energy intake among men, but not among women.
Swan, J. H., et al., 2016. **USA** [75]	Cross-sectional study (administered in face-to-face interviews)	*n* = 989, 60+	To examine the effects of attending Senior Centres (SC), on nutrition and health and efforts to improve diets and weight.	Less than one sixth strongly agreed that their health improved eating at the SC, less than one fourth agreed, whereas more than one third neither agreed nor disagreed. SC attendance and social engagement explained agreement that SC-meals improved nutrition and health but were not shown to predict changes in diet or weight control.
Toner, H. & Morris, D., 1993. **USA** [76]	Cross-sectional study	*n* = 100, 60–83 years	To examine the relationship of self-actualization and nutrition support to dietary intake.	Significant and positive associations between the predictor variables and vitamin A, B vitamin complex, iron and dietary fiber were found. Support from family, friends and neighbors were found to positively influence dietary quality.
Waring, M. L. & Kosberg, J. I., 1984. **USA** [77]	Cross-sectional study	*n* = 55, 60–92 years, median age 64	To investigate the relationship of morale to social and health conditions, level of program participation, and the differential use of social welfare services of the older black people, utilizing a congregate meals program (CMP) in a small town.	CMPs met needs of participants but support services were utilized more than counselling services. Despite that CMPs was used for nourishment and social needs, in was not associated with morale.
Wham, C., et al., 2011. **New Zealand** [42]	Cross-sectional study (administered in face-to-face interviews)	*n* = 51, 80–85 years, mean age 82.4	To assess the nutrition risk status of community living older people and to identify associated risk factors.	A third of the participants (31%) were at high risk of malnutrition, the majority of participants (82%) lived alone and nearly half (47%) had supportive social networks including close relationships with local family, friends and neighbors.
	*Longitudinal*			
Clausen, T., et al., 2007. **Botswana** [50]	Longitudinal population-based study (administered in face-to-face interviews)	*n* = 372, baseline, *n* = 249 follow-up, 60+	To assess diminished function and lack of social support as indicators of short term risk of death.	Overall mortality rate was 10.9 per 100 person years. Age-adjusted odds ratios (OR) for death during follow-up were; 4.2 (CI 1.4–12.5) and 3.6 (CI 1.0–12.7) for those with diminished physical- and cognitive function, respectively. Older community living persons in Botswana with reduced cognitive or physical function, have a significantly increased risk of death.
Lengyel, C. O., et al., 2017. **Canada** [41]	Longitudinal study	*n* = 336 men, mean age 90	To identify patterns of nutritional risk among older men over a four-year period and to project their survival rates over the next two and a half years.	Distinct nutritional risk trajectories were found for older men over a four-year period. Poor nutritional risk trajectories are associated with higher risk of mortality for very old men over a short period of time.
	*Experimental*			
Husted, M. M., et al., 2019. **Denmark** [49]	Single-blinded cluster-controlled study	*n* = 123, 65+ (*n* = 62 intervention group, mean age 82.3; *n* = 61 control group, mean age 83.5)	To understand if older adults have improvement in health-related quality of life, muscle strength, and nutritional status when involved in own meals as part of a rehabilitation program.	There was a significant (*p* = 0.01) improvement of health-related quality of life (converted EQ5D-3L score) in intervention (0.570 vs. 0.668) compared to the control (0.666 vs. 0.580) from baseline to follow-up.
	QUALITATIVE			
Asamane, E. A., et al., 2019. **UK** [78]	Individual interviews (baseline and 8-month follow-up)	*n* = 92, 60+ (baseline *n* = 92, mean age 70.6; 8-month follow-up *n* = 81, mean age 70.7)	To identify and compare factors influencing eating behaviors and physical function among (participants self-identified as) ethnic minorities and understand how these factors and their association with healthy eating and physical function changed over 8 months.	Participants had diverse perceptions of healthy eating and physical function. Healthy eating was viewed as more important than, and unrelated to, physical function. Personal, social and cultural/environmental factors were identified as the main factors influencing these. Eating company were reported to affect eating positively and give greater enjoyment during mealtimes.
Bloom, I., et al., 2017. **UK** [53]	Focus group interviews	*n* = 92, 74–83 years, mean age 78	To explore influences on diet among community-living older people in the UK; and to gain insight into sex differences and factors linked to differences in diet stability in older age.	Age-related factors linked to food choices were lifelong food experiences, retirement, bereavement, medical conditions and environmental factors. Discussion about social activities and isolation, community spirit and loneliness within focus groups, indicated the importance of social engagement as an influence on diet.
Byker Shanks, C., et al., 2017. **USA** [79]	Focus group interviews	n=33, 50+, mean age 73.6	To explore how the rural food environment influences food choices of older adults.	Four themes related to factors influencing food choices emerged: perception of the rural community environment, support as a means of increasing food access, personal access to food sources, and dietary factors.
Cohen, N. & Cribbs, K., 2017. **USA** [55]	Focus group interviews	n=31, 60+	To explore the food practices of LGBT seniors.	Social connection, nostalgia, creativity, material elements and competence came up during discussions. Food practices are entities composed of meanings, materials, and competences that are structured as they are performed repeatedly in a social context.
Falciglia, G., et al., 1985. **USA** [80]	Observations	n=4 older adults	To examine factors of change as they affect older people in four main settings: grocery shopping, meal preparation, meal/snacking patterns and entertainment.	Following factors are identified to effect food changes: health concerns, change in family composition, sensory alterations, income limitations, and social isolation.
Falk, L. W., et al., 1996. **USA** [81]	Individual interviews (2 with each participant)	n=16, 65+	To explain how factors that affect food choice in older people function as food choice processes, and to further theoretical understanding of the food choice process in older adults.	Food choices and preferences were strongly influenced by beliefs related to appropriate food behavior and expected characteristics of foods and meals. Additionally, social context, sensory perceptions, monetary considerations, convenience, and physical well-being.
Foley, E. & BeLue, R., 2017. **Senegal** [57]	Individual interviews	n=41, mean age 58	To identify cultural enablers and barriers to dietary management of type 2 diabetes.	Participants routinely identified the cost of food as a major obstacle to dietary management. Having a different diet or eating separately from the communal family plate creates feelings of social isolation, and reducing servings of traditional foods are described to feel like abandoning a culture.
Hegge, M., 1991. **USA** [51]	Individual interviews	n=26, 60+	Examine problems and coping strategies of newly widowed older people.	Most frequent, troubling problems were loneliness, social isolation, disruption in eating and sleeping patterns. Coping strategies were sense of humor, faith, friends and family.
Howell, B. M. & Bardach, S. H., 2018. **USA** [82]	Individual interviews	*n* = 15, 57–87 years	To identify sociocultural influences on diet and activity patterns for seniors to inform the design of a larger quantitative research project.	Six major themes were identified: the media, friends and peers, family influences, social opportunities, ethnicity and subsistence practices, and weight loss/body weight concerns.
Jager, M. J., et al., 2019. **the Netherlands** [59]	Individual interviews	*n* = 12, 44–87 years	To explore experiences and views of ethnic minority type 2 diabetes patients regarding a healthy diet and dietetic care in order to generate information that may be used for the development of training for dieticians in culturally competent dietetic care.	Respondents acknowledged the importance of a healthy diet. What they considered healthy was determined by culturally influenced ideas about health benefits of specific foods. Social influences were experienced both as supportive and a hindrance.
Knutsen, I. R., et al., 2017. **Five European countries** [58]	Individual interviews	*n* = 125 (*n* = 94, 60+)	To achieve a better understanding of how food is perceived to be significant within persons’ network and relations at different levels, among people with type 2 diabetes.	The respondents′ reflections indicate that there are complex negotiations on different levels that influence self-management and food, including support, knowledge, and relationships within families; attention and openness in social situations; and the premises and norms of society.
Pettigrew, S. & Roberts, M., 2008. **Australia** [52]	Individual interviews	*n* = 19, 65+	To generate specific intervention recommendations, working with loneliness among older adults.	Identified behaviors that ameliorated loneliness: friends and family—emotional resource, engaging in eating and drinking rituals—maintaining social contacts, reading and gardening.
Plastow, N. A., et al., 2015. **UK** [60]	Individual interviews	*n* = 39, 61–89 years, mean age 74	To explore the role of food activities in identity maintenance among community-living older adults.	Two lifelong food identities were discovered: “food lover” and “nonfoodie”. Food activities that are a pleasurable and important part of daily life contribute to the maintenance of important identities and mental well-being in older adults.
Sriram, U., et al., 2018. **USA** [83]	Focus group interviews	*n* = 125, 40–91 years (*n* = 67, 65+)	To explore how social relationships influence health-related behaviors among midlife and older rural adults at increased risk of chronic disease.	Authors found actions and attitudes of family and friends to be key influences on diet, physical activity, and tobacco use behaviors, positively and negatively. Older women expressed that loneliness was a barrier for healthy eating, due to lack of motivation to prepare healthy meals. “Food-centric events”, including shared meals, also expressed by both older and middle aged persons as part of peer influences on diet.
Tessier, S. & Gerber, M., 2005. **Sardinia/Malta** [84]	Individual interviews	*n* = 30 mother-daughter couples, mean age 66 and 39, respectively	To compare meals, in-between meals snack consumption and total daily food intakes between Sardinia and Malta in terms of structure, social environment and times, as well as their changes.	There were striking contrasts between Sardinian and Maltese food habits, such as meal preparation times, breakfast and main meal structures, total daily food intake profiles and commensality.
Thompson, J., et al., 2017. **UK** [85]	Individual interviews	*n* = 20 men, 65–90 years, mean age 80	To understand the potential for issues around food vulnerability to arise in older bereaved men and to characterize that vulnerability, if present.	Five overarching themes were identified: financial security, social networks, cooking skills, food and routine and single servings.
Uribe, A. C. R., 2019. **Mexico** [86]	Individual interviews	*n* = 14 women, 64–87 years, mean age 76	To explain the cooking and eating behaviors of Mexican older women living alone using a life course perspective	Ten out of fourteen reported finding eating alone not enjoyable at all. It used to be a time for socializing with families, sharing, and having fun. Coping strategies included reading, watching television, listening to music, and inviting friends and neighbors over for shared meals. A table for one was also said to result in changed eating habits, for example by skipping meals, and eating more while cooking less.
Whitelock, E. & Ensaff, H., 2018. **UK** [54]	Focus group interviews	*n* = 30, 63–90 years	To explore older adults’ perceptions and practices related to dietary behavior and the factors influencing their food choice in later life.	Age-related changes, food access, on your own and relationship with food are themes that emerged where living alone and social isolation often were discussed.
	MIXED DESIGN			
Gustafsson, K. & Sidenvall, B., 2002. **Sweden** [87]	Individual interviews and 3-day food diary	*n* = 18 women, 65–88 years	To explore food-related health perceptions and food habits among older women.	The first theme “a healthy slimming meal or the usual” summarized the women′s health perceptions related to food, where the dominating view was fear of fat. The second theme “meals—a pleasure or an obligation” showed that meals in fellowship were perceived as a pleasure, and women living alone tended to simplify cooking and eating.
Hughes, G., et al., 2004. **UK** [88]	Cross-sectional survey and individual interviews	*n* = 39 men, 62–94 years, mean age 74.8	To investigate barriers to healthy eating, focusing on energy intake, food choice (specifically fruits and vegetables), cooking skills and well-being in a group of older men living alone.	BMI failed to predict patterns of intake. Men with good cooking skills reported better physical health and higher intake of vegetables. Interviews revealed that poor cooking skills and low motivation to change eating habits may constitute barriers to improving energy intake, healthy eating and appetite.
Johnson, C.S., 2002. **Canada** [56]	Cross-sectional survey and focus group interviews	*n* = 22, 60–85 years, mean age 72	To examine level of nutritional risk and dietary issues faced by older adults in three groups: recently bereaved, with or without intervention and those in coupled relationships.	Bereaved individuals had moderate risk for poor nutrition, irrespectively if they had counselling for grief resolution or not. Those in coupled relationships had the lowest risk. One dietary issue for bereaved individuals was memories of shared meals, coping by changing setting (ex in front of TV) or inviting friends/family.
Shifflett, P.A., 1987. **USA** [89]	Observations, individual interviews and 7-day food record	*n* = 30, 60–91 years	To investigate the process of negotiating food habit changes among older people, including future time perspective and past experiences.	Future time perspective and past experiences were significant in negotiating process. Changes were either externally motivated (physician-prescribed) or internally motivated (self-prescribed).
Zelig, R., et al., 2019. **USA** [90]	Individual interviews and nutritional assessment	*n* = 19, 66–83 years, mean age 71.3	To explore the eating experience and eating-related quality of life (ERQOL) of community-living older adults with tooth loss.	Some participants ate before going out or avoided eating out due to embarrassment, others had no embarrassment or self-consciousness. Participant at risk for malnutrition more frequently reported that eating got harder over time and had less social interaction, less enjoyment from food, more food avoidance, and more embarrassment related to their tooth loss.

## 4. Discussion

Having mapped out scientific articles presently available on eating alone or with others among community-living older people, we found a wide range of topics and methods. Research on nutritional status, depression, household eating arrangements, loneliness, and coping with grief are common topics. Furthermore, the studies consisted of both quantitative and qualitative study designs, such as cross-sectional surveys, experimental studies, qualitative interviews, and participant observations. In about one-third of the articles, eating alone or together are treated as central topics of interest, isolated from adjoining and broader concepts such as social participation, social activities, or social isolation. In about half, eating alone or together are one aspect of the findings, for example, treated as one of several variables in a regression model, one of several identified risk factors for malnutrition or depression, or as one of several significant aspects of social life in general. Summarized, our findings confirm the significance of commensality in community-living older peoples’ lives—in several cultural and geographical contexts, with different household compositions, and with diverse health conditions. Aspects that were highlighted less than one might anticipate from a public health perspective were socioeconomic factors and gender. A few studies highlight income as a relevant variable [13,23,64,80] and gender differences of eating alone are specifically examined in four studies included [15,21,53,74]. Moreover, some studies show associations between eating alone and negative health outcomes in men but not in women, regarding depressive symptoms [21], metabolic syndrome [14], and mortality [15,16]. These are aspects that could benefit from further analyses. However, based on our findings, we argue that the main gaps identified in the literature are two of a different kind: study designs for finding causal relationships and an improved differentiation in the measurements of eating alone or with others.

First, even though our method provides no information about methodological quality of individual studies, or combined effects of the strength of the evidence for any given relationship, we can see that the main results of quantitative studies tend to point in one direction—eating alone is suggested to have negative effects on the target population—and they are dominated by cross-sectional studies. High-quality randomized controlled trials are lacking and longitudinal studies are few; thus, no claims of causality can be made. For example, a cross-sectional study cannot rule out if meals eaten alone is a common feature of a person’s life before or after feelings of loneliness or depressive symptoms occur. Nor can we say, for example, that malnutrition is caused by the lack of meal company, or if the causality is reversed (e.g., people with eating problems avoiding shared meals). In contrast, qualitative studies provide richness and nuances to large-scale statistical patterns. Talk about reasons for low weight [30] or changes in food behaviors due to the loss of commensality [32], for example, rely on subjective recollections from small groups of people. Such studies are indeed important but must be interpreted with their methodological limitations in mind. Moreover, different forms of mealtime interventions have previously targeted institutionalized older people [8,91] but are clearly lacking for the community-living population. Institutionalized older people might be easier to include in an intervention, simply because it is possible to target a specific hospital or care home, and this is no doubt a relevant target group. However, as both quantitative and qualitative studies included in this article suggest, some community-living older people could possibly benefit from mealtime interventions as well, for example to improve nutritional status or prevent feelings of loneliness. The pilot study by McHugh Power et al. [27] is an example of a design that could inspire further RCTs, as does the cluster-controlled study by Husted et al. [49]. Such studies should be pre-registered and designed with the ambition of identifying, for example, whether sharing meals may have clear causal effects on health outcomes (e.g., nutritional status, general well-being, social isolation, etc.) and, if so, whether this is fundamentally different from the effects of other social activities. Regarding the scarcity of longitudinal data, prospective cohort studies could add questions pertaining to eating alone or with others, but this must be done with caution, which brings us to our next point.

Second, as the results show, measurements of eating alone or with others are often created in similar ways, with people responding if they eat alone, how often, and possibly with whom. This is a stark contrast to the literature on loneliness, in which significant work has been put into classification and measurement in order to capture the nuances of what it means to be “alone”, e.g., [92,93]. This literature conveys that questions about how many people one is sharing a household with, or how many friends one has—objective loneliness—are inadequate to capture the subjective dynamics of loneliness. We suggest that researchers trying to measure commensality search for inspiration in this literature. We also suggest that measurements of commensality should be informed by qualitative findings, since these, as our results show, may add complexity and nuances from stories of older people who are neutral about having eating companions, or actually prefer eating alone.

### Strengths and Limitations

One important advantage of a scoping review, including this one, is its comprehensive mapping of a very broad range of studies on a given topic, providing a summary view of what has been done and where important gaps may be. This strength of the comprehensiveness must, however, be weighed against the limitations of not evaluating the quality of individual studies or make qualitative judgments about the strength of the evidence of a relationship between a given exposure and outcome, as in systematic reviews [94]. Nor can we draw any combined statistical conclusions from the quantitative studies, which we could have done with a meta-analysis. However, this is not the aim of a scoping review. Our general mapping of the literature can hopefully stimulate such future endeavors.

A strength of this particular scoping review is that it covers a wide range of research from geographical regions, academic disciplines, and methodologies. Two reviewers checked all of the abstracts independently; moreover, the screening of full-texts was a collaborative effort of continuous author deliberation, with generous criteria for inclusion. Furthermore, we added publications found in reference lists of relevant publications. We therefore see it as unlikely that publications that could substantially alter our findings were overlooked.

The broadness of our findings provides us with an understanding of both the scientific breadth of the questions as well as the cross-cultural significance in peoples’ lives. However, this also makes it difficult to collate them and abstract key concepts from the findings. Moreover, although the findings are broad, our review only includes studies focused on eating. This means, for example, that the social aspects of cooking, also acknowledged as meaningful in sociological research, e.g., [95,96], were excluded. The search method also means that some commensality research on social sciences and humanities not connected to such databases may have been overlooked (e.g., book chapters or articles in non-indexed journals). Lastly, scoping reviews may include “gray literature” [10], and our findings could perhaps have been more informative had we done the same. Our aim, nevertheless, was explicitly to map out scientific publications for the purposes of scientific research; hence, gray literature was considered redundant.

## 5. Conclusions

Findings from this scoping review demonstrate that social aspects of eating are acknowledged by several researchers and research teams, from a variety of research fields and countries. However, how close this question is to the core of a given study varies, and the majority of articles do not have eating alone or together as its central topic. We show a wide range of topics and methods, with relatively similar patterns regarding the main results, but also with scientifically important exceptions. Based on our findings we recommend future research designs that allow for the identification of causal relationships, use more refined ways of measuring meals alone or together, as well as qualitative methods that add complexity and nuances to the explored phenomena.

## Data Availability

No new data were created or analyzed in this study. Data sharing is not applicable to this article.

## Supplementary Materials

The following are available online at https://www.mdpi.com/article/10.3390/ijerph18073495/s1, Table S1: Databases and search terms; Table S2: Documentation of full electronic search strategy; Table S3: Articles in the third category: peripheral; Table S4: Geographical spread of included articles.

## Appendix A

**Table A1:** Databases and search terms.

**Databases:** PubMed, Web of Science, Cinahl and PsycInfo.		
**Block 1: Target** **G** **roup**	**Block 2: Meals**	**Block 3: Social Aspects**
Old age	Commensality	Social Engagement
Old people	Meals	Social Participation
Older people	Eating	Social Interaction
Old adults	Diet	Social Norm(s)
Older adults	Eating alone	Social Context
Elderly	Food intake	Social Environment
Aging (Only MeSH)	Dietary intake	Social Isolation
Aged (Only MeSH)	Food choice	Social Network(s)
	Food habits	Social Influence
	Social eating	Social Facilitation
	Eating behavior	Social Modeling
	Food consumption	Single living
	Solo dining	Living alone
	Solo eating	Social Isolation (Only MeSH)
	Food (Only MeSH)	
	Meals (Only MeSH)	

## Appendix B

**Table A2:** Documentation of full electronic search strategy (Example from PubMed).

*Search Number*	Block	Search Terms
***1***	Target group	(((((((Elderly[Title/Abstract]) OR (“Old age”[Title/Abstract])) OR (“Old people”[Title/Abstract])) OR (“Older people”[Title/Abstract])) OR (“Old adults”[Title/Abstract])) OR (“Older adults”[Title/Abstract])) OR (aging[MeSH Terms])) OR (aged[MeSH Terms])
***2***	Meals	(((((((((((((((Commensality[Title/Abstract]) OR (Meals[Title/Abstract])) OR (Eating[Title/Abstract])) OR (Diet[Title/Abstract])) OR (Eating alone[Title/Abstract])) OR (Food intake[Title/Abstract])) OR (Dietary intake[Title/Abstract])) OR (Food choice[Title/Abstract])) OR (Food habits[Title/Abstract])) OR (“Social eating“[Title/Abstract])) OR (“Eating behavior”[Title/Abstract])) OR (“Food consumption”[Title/Abstract])) OR (“Solo dining”[Title/Abstract])) OR (“Solo eating”[Title/Abstract])) OR (food[MeSH Terms])) OR (meals[MeSH Terms])
***3***	Social aspects	(((((((((((((((Social Engagement[Title/Abstract]) OR (Social Participation[Title/Abstract])) OR (Social Interaction[Title/Abstract])) OR (Social Norm[Title/Abstract])) OR (Social Norms[Title/Abstract])) OR (“Social Context”[Title/Abstract])) OR (“Social Environment”[Title/Abstract])) OR (“Social Isolation”[Title/Abstract])) OR (“Social Network”[Title/Abstract])) OR (“Social Networks”[Title/Abstract])) OR (“Social Influence”[Title/Abstract])) OR (“Social Facilitation”[Title/Abstract])) OR (“Social Modeling”[Title/Abstract])) OR (“Single living”[Title/Abstract])) OR (“Living alone”[Title/Abstract])) OR (social isolation[MeSH Terms])
***4***	Combined	1 AND 2 AND 3

## Appendix C

### Appendix C.1. The Third Category: Eating Alone is Peripheral

This category has the smallest number of articles (*n* = 18). Due to the peripheral nature of the studies, these are not described in depth, although some examples are mentioned in order to clarify how they are “peripheral” and still included (Table A3).

### Appendix C.2. Quantitative Studies

Three studies focusing on cancer patients used a questionnaire, in combination with a cancer specific scale or module [97,98,99]. Looking into the cancer specific scale, the “Eating in public subscale” was found to include a question about eating with others [97,98]. Another cancer specific module included seven multi-item symptom scales where one was mentioned as “social eating” [99]. The results of the Eating in public subscale and the symptom scale for social eating is peripheral in the articles. In other words, eating alone or with others has a minor part of the design but is neither analyzed nor touched upon in the articles.

In other studies, questions were asked about eating with others but not mentioned in the article [100,101] or only indirectly touched upon through a variable of “going out to eat” [102] or with “social interaction” as a mentioned reason for older people to participate in congregate meal programs [103].

### Appendix C.3. Qualitative Studies

As a qualitative example, a focus group interview study explored built and social environmental facilitators of, and barriers to, engagement in eating a healthy diet, physical activity, and regular contact with other people, among older Chinese immigrants in Australia [104]. One part of their findings was that lack of social support from family members was a barrier to a healthy diet, further the participants mention eating in restaurants or community centers but did not describe the eating company or lack thereof.

**Table A3:** Articles included in the third category: peripheral.

Reference	Study Design	Participants	Aim	Result
	QUANTITATIVE			
	*Cross-sectional*			
Allal, A. S., et al., 2000. **Switzerland** [97]	Cross-sectional study	*n* = 21 head and neck cancer patients, 40+, median age 63	To evaluate quality of life (QOL) and functional outcome in patients with carcinomas of the larynx and hypopharynx treated with accelerated radiotherapy (RT).	The PSSHN scores were 89, 84, and 86, respectively, for eating in public, understandability of speech and normalcy of diet (100 = normal function). QOL and functional outcome in patients treated conservatively with concomitant boost RT for *laryngeal and hypopharyngeal carcinomas* appear to be similar to those obtained in patients treated with conventional or hyperfractionated RT.
Allal, A. S., et al., 2003. **Switzerland** [98]	Cross-sectional study	*n* = 60 head and neck cancer patients, 42+, median age 61	To assess quality of life (QoL) outcomes of patients after two treatment strategies: radical surgery with postoperative RT and accelerated concomitant boost RT with or without chemotherapy.	The PSSHN scores were generally higher in the RT group, with a significant difference in the speech subscale (*p* = 0.005), a trend for a significant difference for the eating in public subscale (*p* = 0.08), and an insignificant difference for the normalcy of diet subscale (*p* = 0.25). For early stages no clear advantage in QoL outcome was noted for the RT group compared with the surgery group, for advanced-stage disease an advantage favoring radical RT seemed apparent.
Byeon H., 2019. **South Korea** [105]	Cross-sectional study	*n* = 142, 65+	To identify factors affecting the swallowing quality-of-life of older people.	33.9% of the elderly had low swallowing quality-of-life. The proportion of the elderly with a low swallowing quality-of-life was significantly higher when the subjects were equal to or older than 75 years old (38.4%), were female (44.6%), were elementary school graduate or below (35.7%) and were living alone (31.7%).
Hoerster, K. D., et al., 2016. **USA** [100]	Cross-sectional study	*n* = 653, 25–97 years, mean age 60.8	To examine individual, social environment, and physical environment correlates of general diet quality among Veterans.	Higher level of depressive symptom severity, not having others to eat healthy meals with and reduced availability of low-fat foods in stores were associated with poorer diet quality.
Kjaer, T. K., et al., 2016. **Denmark** [99]	Cross-sectional study	*n* = 369 head and neck cancer survivors, mean age 64 (*n* = 234, 60+)	To investigate associations between social factors, length of education and cohabitation status, and the occurrence of clinically relevant, patient-reported late effects, with control for important disease-related factors, such as site, stage, and HPV status, and treatment and lifestyle factors and comorbidity.	Survivors with short education were more likely to report severe problems than those with medium or long education. For survivors who lived alone, the adjusted ORs were significantly increased for physical functioning (2.17; 95% CI 5 1.01–4.68) and trouble with social eating (OR 5 2.26; 95% CI 5 1.14–4.47).
Lai, X. J., et al., 2019. **China** [106]	Cross-sectional study	*n* = 290 retired couples and *n* = 1.571 working couples	To investigate how retirement would change their activity time use and patterns. In particular, intra-household interactions are considered, to explore the interdependencies among household members’ choices, social-demographics and travel behaviors.	Retirement would substantially increase joint participations and durations in various out-of-home activities. In addition, the importance of walkability is emphasized for retired couples in a mixed-land-use and transit-dependent city, and a potential social exclusion issue is identified for the low-income retired population.
Quandt, S. A. & Rao, P., 1999. **USA** [107]	Cross-sectional study (administered in face-to-face interviews)	*n* = 192, 65+	To asses level of food insecurity and identify predictors.	24% report one or more food insecurity indicator. Eating alone is together with taking three or more prescription drugs and income less than 150% of poverty level predictors of food insecurity.
Wham, C. A., et al., 2015. **New Zealand** [101]	Cross-sectional study	*n* = 255 Māori and *n* = 400 non-Māori adults, 80–90 years	To establish the prevalence of high nutrition risk and associated health and social risk factors for New Zealand Māori and non-Māori participants.	Half (49%) of Māori and 38% of non-Māori participants were at high nutrition risk (SCREEN II score <49). For non- Māori high nutrition risk was associated with female sex (*p* = 0.005), living alone (*p* = 0.002), a lower physical health related quality of life (*p* = 0.02) and depressive symptoms (*p* = 0.002).
	*Longitudinal*			
Ang, S., 2018. **Singapore** [102]	Longitudinal study (administered in face-to-face interviews)	*n* = 4.482, mean age men 71.6;mean age women 72.2	Investigates if ethnicity accounts for sex differences in (a) the types of social activities older adults participate in and (b) the association between social participation and 4-year mortality.	Men were more likely to engage in social activities compared to women, sex difference varied by ethnicity. Going out to eat was associated with a lower risk of mortality for men only and playing sports was found to be protective for women only.
Arcury, T. A., et al., 2012. **USA** [108]	Longitudinal study (administered in face-to-face interviews, 2 with each participant and one month interval)	*n* = 593 (first interview); *n* = 563 (second interview) 60+	To describe diabetes management behaviors and social integration among older adults, and delineate the associations of social integration with diabetes management behaviors.	Participants had high levels of social integration and largely adhered to diabetes management behaviors. Social integration was associated with social network size, particularly other relatives seen and spoken with on the telephone among others.
Mamalaki, E., et al., 2019. **Greece** [109]	Longitudinal cohort study	*n* = 1.933 adults, 65–99 years, mean age 73.1	To explore the associations between social life and adherence to a healthy dietary pattern, the Mediterranean diet (MD), in a population-representative cohort of older people.	Each unit increase in the number of social contacts/month and in the frequency score of intellectual, social and physical activities was associated with a 1·6, 6·8, 4·8 and 13·7% increase in the likelihood of a participant being in the high MD adherence group, respectively.
	QUALITATIVE			
Cerin, E., et al., 2019. **Australia** [104]	Focus group interviews	*n* = 91, 60–85 years, mean age 71.1	To identify built and social environmental facilitators of and barriers to regular engagement in physical activity, eating a healthy diet and regular contact with other people among older Chinese immigrants to urban Australia.	For physical activity the highest ranked facilitator and barrier was “proximity to destinations” and “poor/inadequate public transport”, respectively. For healthy diet the highest ranked facilitator and barrier were “high food safety standards/regulations” and “lack of family/household members′ social support for a healthy diet”.
Lee, K.-I., 2009. **USA** [103]	Focus group interviews	*n* = 39, 62–92 years, mean age 78	To explore community-living elderly beliefs for participating in congregate meal programs and to identify salient beliefs by category (behavioral beliefs, normative beliefs and control beliefs).	Advantages of participating are nutrition implication, social interaction, special diets, low price, convenience and less waste.
van den Heuvel, E., et al., 2018. **UK** [110]	Individual and focus group interviews	*n* = 42, 56–96 years, mean age 67	To explore all reasons for consuming and not consuming eggs in older adults.	Thematic analyses revealed 69 different reasons for eating or not eating eggs and were related to: properties of the food, convenience, medical factors and social environment. The category “Social environment” included social influence of anyone present at an eating occasion.
Zou, P., 2019. **Canada** [111]	Individual interviews	*n* = 30, mean age 61	To determine the facilitators and barriers influencing healthy eating behaviors among aged Chinese-Canadians with hypertension.	The analyze resulted in personal, familial, community and societal factors. Among “Familial factors” on diet, other people’s preferences and habits was mentioned. For example, that it is easier to cook healthy in a small family, with only two people sharing a meal. Being alone could also affect motivation to cook negatively.
	MIXED DESIGN			
Glover, L., et al., 2020. **UK** [112]	Co-creation study, 4 meetings	*n* = 10 lay people, 70–79 years and *n* = 4 university members, 27–57 years	To undertake a co-creation study, explore maintenance of health and well-being in older age, and the application of co-creation with an older community population and evaluate the process to inform future work.	Findings demonstrate that state of mind and of health were key to well-being in older age. Feeling safe, comfortable and pain free were important along with being able to adapt to change, have choice and a sense of personal freedom. Social connectedness was seen as the keystone to support healthy behaviors.
Howell, B. M., 2020. **USA** [113]	Cross-sectional survey, participant observation and individual interviews	*n* = 82, 65+, mean age 74	To examine the relationship between the sociocultural factors that shape diet, physical activity, and nutritional status outcomes among seniors.	T-tests indicate that diet and physical activity practices in this sample do not meet national recommendations and that diet differs adversely from national reference samples. Family influences increased fruit consumption, while participation in cultural and social events increased intake of fats and sweets.
Nogueira, D., et al., 2019. **Portugal** [114]	Three phase evaluation study	*n* = 105 patients (with dysarthria), 18–102 years, mean age 64; and *n* = 103 control (without), 18–87, mean age 50.6	To produce a European Portuguese version of the Quality of Life Questionnaire for the Dysarthric Speaker (QoL-DyS).	The QoLDyS correlated positively with the QAD, PEAT-10, and EQ5D. Cronbach′s α was 0.973, and it remained excellent when any item was deleted.

## Appendix D

**Table A4:** Geographical spread of included articles (*n* = 72), also distributed in respective categories. The geographical origin of articles was divided in accordance with the regional classification of the United Nations [115].

Geographic Regions	Total	Central Topic	One Aspect	Peripheral
*Northern Africa*	0 (0%)			
*Sub-Saharan Africa*	2 (2%)		2	
*Northern America*	39 (40%)	7	26	6
*Caribbean*	0 (0%)			
*Central America*	1 (1%)		1	
*Latin America*	0 (0%)			
*Central Asia*	0 (0%)			
*Eastern Asia*	16 (16%)	13	1	2
*South-Eastern Asia*	0 (0%)			
*Southern Asia*	1 (1%)			1
*Western Asia*	3 (3%)	1	2	
*Northern Europe*	23 (24%)	8	12	3
*Eastern Europe*	0 (0%)			
*Southern Europe*	4 (4%)		2	2
*Western Europe*	4 (4%)		2	2
*Oceania*	5 (5%)	1	2	2
	98 (100%)	30	50	18
