# Supplementary material for: Eating Alone or Together among Community-Living Older People—A Scoping Review
Source: Int J Environ Res Public Health. 2021 Mar 27;18(7):3495. doi: 10.3390/ijerph18073495 (PMC8036467; doi:10.3390/ijerph18073495)
Supplement: Supplementary file 1 [file ijerph-18-03495-s001.zip › Appendix A.docx]

APPENDIX 1

Table 3 Databases and search terms

| Databases: PubMed, Web of Science, Cinahl and PsycInfo  Block 1: Target group Block 2: Meals Block 3: Social aspects | | |
| --- | --- | --- |
| Old age | Commensality | Social Engagement |
| Old people | Meals | Social Participation |
| Older people | Eating | Social Interaction |
| Old adults | Diet | Social Norm(s) |
| Older adults | Eating alone | Social Context |
| Elderly | Food intake | Social Environment |
| Aging (Only MeSH) | Dietary intake | Social Isolation |
| Aged (Only MeSH) | Food choice | Social Network(s) |
|  | Food habits | Social Influence |
|  | Social eating | Social Facilitation |
|  | Eating behavior | Social Modeling |
|  | Food consumption | Single living |
|  | Solo dining | Living alone |
|  | Solo eating | Social Isolation (Only MeSH) |
|  | Food (Only MeSH) |  |
|  | Meals (Only MeSH) |  |
