# Supplementary material for: Eating Alone or Together among Community-Living Older People—A Scoping Review
Source: Int J Environ Res Public Health. 2021 Mar 27;18(7):3495. doi: 10.3390/ijerph18073495 (PMC8036467; doi:10.3390/ijerph18073495)
Supplement: Supplementary file 1 [file ijerph-18-03495-s001.zip › Appendix B.docx]

APPENDIX 2

Table 4 Documentation of full electronic search strategy (Example from PubMed)

| Search number | Block | Search terms |
| --- | --- | --- |
| **1** | Target group | (((((((Elderly[Title/Abstract]) OR (“Old age”[Title/Abstract])) OR (“Old people”[Title/Abstract])) OR (“Older people”[Title/Abstract])) OR (“Old adults”[Title/Abstract])) OR (“Older adults”[Title/Abstract])) OR (aging[MeSH Terms])) OR (aged[MeSH Terms]) |
| **2** | Meals | (((((((((((((((Commensality[Title/Abstract]) OR (Meals[Title/Abstract])) OR (Eating[Title/Abstract])) OR (Diet[Title/Abstract])) OR (Eating alone[Title/Abstract])) OR (Food intake[Title/Abstract])) OR (Dietary intake[Title/Abstract])) OR (Food choice[Title/Abstract])) OR (Food habits[Title/Abstract])) OR (“Social eating“[Title/Abstract])) OR (“Eating behavior”[Title/Abstract])) OR (“Food consumption”[Title/Abstract])) OR (“Solo dining”[Title/Abstract])) OR (“Solo eating”[Title/Abstract])) OR (food[MeSH Terms])) OR (meals[MeSH Terms]) |
| **3** | Social aspects | (((((((((((((((Social Engagement[Title/Abstract]) OR (Social Participation[Title/Abstract])) OR (Social Interaction[Title/Abstract])) OR (Social Norm[Title/Abstract])) OR (Social Norms[Title/Abstract])) OR (“Social Context”[Title/Abstract])) OR (“Social Environment”[Title/Abstract])) OR (“Social Isolation”[Title/Abstract])) OR (“Social Network”[Title/Abstract])) OR (“Social Networks”[Title/Abstract])) OR (“Social Influence”[Title/Abstract])) OR (“Social Facilitation”[Title/Abstract])) OR (“Social Modeling”[Title/Abstract])) OR (“Single living”[Title/Abstract])) OR (“Living alone”[Title/Abstract])) OR (social isolation[MeSH Terms]) |
| **4** | Combined | 1 AND 2 AND 3 |
