# Supplementary material for: Eating Alone or Together among Community-Living Older People—A Scoping Review
Source: Int J Environ Res Public Health. 2021 Mar 27;18(7):3495. doi: 10.3390/ijerph18073495 (PMC8036467; doi:10.3390/ijerph18073495)
Supplement: Supplementary file 1 [file ijerph-18-03495-s001.zip › Appendix C.docx]

APPENDIX 3

*The third category: Eating alone is peripheral*

This category has the smallest number of articles (n=19). Due to the peripheral nature of the studies, these are not described in depth, although some examples are mentioned in order to clarify how they are “peripheral” and still included (Table 3).

*3.4.1 Quantitative studies*

Three studies focusing on cancer patients used a questionnaire, in combination with a cancer specific scale or module [96-98]. Looking into the cancer specific scale, the “Eating in public subscale” was found to include a question about eating with others [96,97]. Another cancer specific module, included seven multi-item symptom scales where one was mentioned as “social eating” [98]. The results of the Eating in public subscale and the symptom scale for social eating is peripheral in the articles. In other words, eating alone or with others has a minor part of the design but is neither analyzed nor touched upon in the articles.

In other studies, questions were asked about eating with others but not mentioned in the article [99,100] or only indirectly touched upon through a variable of “going out to eat” [101] or with “social interaction” as a mentioned reason for older people to participate in congregate meal programs [102].

*3.4.2 Qualitative studies*

As a qualitative example, an interview study with older participants recovering from a coronary heart disease [103] was included in this category. A peripheral part of the article concerned meals in company with others or alone, in which participants indicated that cooking for one could feel like “it’s too much work for one person” [103] (p. 267) but it was unclear whether this had anything to do with the social company as such.

Table 5 Articles in the third category: peripheral

| **Reference** | **Study design** | **Participants** | **Aim** | **Result** |
| --- | --- | --- | --- | --- |
|  | **Quantitative** |  |  |  |
| Allal, A. S., et al. 2000. **Switzerland** [96] | Cross-sectional study | n=21 head and neck cancer patients, 40+, median age 63 | To evaluate quality of life (QOL) and functional outcome in patients with carcinomas of the larynx and hypopharynx treated with accelerated radiotherapy (RT). | The PSSHN scores were 89, 84, and 86, respectively, for eating in public, understandability of speech and normalcy of diet (100 = normal function). QOL and functional outcome in patients treated conservatively with concomitant boost RT for *laryngeal and hypopharyngeal carcinomas* appear to be similar to those obtained in patients treated with conventional or hyperfractionated RT. |
| Allal, A. S., et al. 2003. **Switzerland** [97] | Cross-sectional study | n=60 head and neck cancer patients, 42+, median age 61 | To assess quality of life (QoL) outcomes of patients after two treatment strategies: radical surgery with postoperative RT and accelerated concomitant boost RT with or without chemotherapy. | The PSSHN scores were generally higher in the RT group, with a significant difference in the speech subscale (p = .005), a trend for a significant difference for the eating in public subscale (p = .08), and an insignificant difference for the normalcy of diet subscale (p = .25). For early stages no clear advantage in QoL outcome was noted for the RT group compared with the surgery group, for advanced-stage disease an advantage favoring radical RT seemed apparent. |
| Ang, S. 2018. **Singapore** [101] | Longitudinal study (administered in face-to-face interviews) | n=4.482, mean age men 71.6;mean age women 72.2 | Investigates if ethnicity accounts for gender differences in (a) the types of social activities older adults participate in and (b) the association between social participation and 4-year mortality. | Men were more likely to engage in social activities compared to women, gender difference varied by ethnicity. Going out to eat was associated with a lower risk of mortality for men only and playing sports was found to be protective for women only. |
| Arcury, T. A., et al. 2012. **USA** [104] | Longitudinal study (administered in face-to-face interviews, 2 with each participant and one month interval) | n=593 (first interview); n=563 (second interview) 60+ | To describe diabetes management behaviors and social integration among older adults, and delineate the associations of social integration with diabetes management behaviors. | Participants had high levels of social integration and largely adhered to diabetes management behaviors. Social integration was associated with social network size, particularly other relatives seen and spoken with on the telephone among others. |
| Byeon H. 2019. **South Korea** [105] | Cross-sectional study | n=142, 65+ | To identify factors affecting the swallowing quality-of-life of older people. | 33.9% of the elderly had low swallowing quality-of-life. The proportion of the elderly with a low swallowing quality-of-life was significantly higher when the subjects were equal to or older than 75 years old (38.4%), were female (44.6%), were elementary school graduate or below (35.7%) and were living alone (31.7%). |
| Hoerster, K. D., et al. 2016. **USA** [99] | Cross-sectional health behavior study | n=653, 25-97 years, mean age 60.8 | To examine individual, social environment, and physical environment correlates of general diet quality among Veterans. | Higher level of depressive symptom severity, not having others to eat healthy meals with and reduced availability of low-fat foods in stores were associated with poorer diet quality. |
| Kjaer, T. K., et al. 2016. **Denmark** [98] | Cross-sectional study | n=369 head and neck cancer survivors, mean age 64 (n=234, 60+) | To investigate associations between social factors, length of education and cohabitation status, and the occurrence of clinically relevant, patient-reported late effects, with control for important disease-related factors, such as site, stage, and HPV status, and treatment and lifestyle factors and comorbidity. | Survivors with short education were more likely to report severe problems than those with medium or long education. For survivors who lived alone, the adjusted ORs were significantly increased for physical functioning (2.17; 95% CI 5 1.01–4.68) and trouble with social eating (OR 5 2.26; 95% CI 5 1.14–4.47). |
| Lai, X. J., et al. 2019. **China** [106] | Cross-sectional study | n=290 retired couples and n=1.571 working couples | To investigate how retirement would change their activity time use and patterns. In particular, intra-household interactions are considered, to explore the interdependencies among household members’ choices, social-demographics and travel behaviors. | Retirement would substantially increase joint participations and durations in various out-of-home activities. In addition, the importance of walkability is emphasized for retired couples in a mixed-land-use and transit-dependent city, and a potential social exclusion issue is identified for the low-income retired population. |
| Mamalaki, E., et al. 2019. **Greece** [107] | Longitudinal cohort study | n=1.933 adults, 65-99 years, mean age 73.1 | To explore the associations between social life and adherence to a healthy dietary pattern, the Mediterranean diet (MD), in a population-representative cohort of older people. | Each unit increase in the number of social contacts/month and in the frequency score of intellectual, social and physical activities was associated with a 1·6, 6·8, 4·8 and 13·7 % increase in the likelihood of a participant being in the high MD adherence group, respectively. |
| Quandt, S. A., & Rao, P. 1999. **USA** [108] | Cross-sectional study (administered in face-to-face interviews) | n=192, 65+ | To asses level of food insecurity and identify predictors. | 24% report one or more food insecurity indicator. Eating alone is together with taking three or more prescription drugs and income less than 150% of poverty level predictors of food insecurity. |
| Wham, C. A., et al. 2015. **New Zealand** [100] | Cross-sectional study | n=255 Māori and n=400 non-Māori adults, 80-90 years | To establish the prevalence of high nutrition risk and associated health and social risk factors for New Zealand Māori and non-Māori participants. | Half (49%) of Māori and 38% of non-Māori participants were at high nutrition risk (SCREEN II score <49). For non- Māori high nutrition risk was associated with female gender (p=0.005), living alone (p=0.002), a lower physical health related quality of life (p=0.02) and depressive symptoms (p=0.002). |
|  | **Qualitative** |  |  |  |
| Cerin, E., et al. 2019. **Australia** [109] | Focus group interviews | n=91, 60-85 years, mean age 71.1 | To identify built and social environmental facilitators of and barriers to regular engagement in physical activity, eating a healthy diet and regular contact with other people among older Chinese immigrants to urban Australia. | For physical activity the highest ranked facilitator and barrier was “proximity to destinations” and “poor/inadequate public transport”, respectively. For healthy diet the highest ranked facilitator and barrier were “high food safety standards/regulations” and “lack of family/household members’ social support for a healthy diet”. |
| Lee, K.-I. 2009. **USA** [102] | Focus group interviews | n=39, 62-92 years, mean age 78 | To explore community-living elderly beliefs for participating in congregate meal programs and to identify salient beliefs by category (behavioral beliefs, normative beliefs and control beliefs). | Advantages of participating are nutrition implication, social interaction, special diets, low price, convenience and less waste. |
| van den Heuvel, E., et al. 2018. **UK** [110] | Individual and focus group interviews | n=42, 56-96 years, mean age 67 | To explore all reasons for consuming and not consuming eggs in older adults. | Thematic analyses revealed 69 different reasons for eating or not eating eggs and were related to: properties of the food, convenience, medical factors and social environment. The category "Social environment" included social influence of anyone present at an eating occasion. |
| Zou, P. 2019. **Canada** [111] | Individual interviews | n=30, mean age 61 | To determine the facilitators and barriers influencing healthy eating behaviors among aged Chinese-Canadians with hypertension. | The analyze resulted in personal, familial, community and societal factors. Among "Familial factors" on diet, other people's preferences and habits was mentioned. For example, that it is easier to cook healthy in a small family, with only two people sharing a meal. Being alone could also affect motivation to cook negatively. |
|  | **Mixed** |  |  |  |
| Glover, L., et al. 2020. **UK** [112] | Co-creation study, 4 meetings | n=10 lay people, 70-79 years and n=4 university members, 27-57 years | To undertake a co-creation study, explore maintenance of health and well-being in older age, and the application of co-creation with an older community population and evaluate the process to inform future work. | Findings demonstrate that state of mind and of health were key to well-being in older age. Feeling safe, comfortable and pain free were important along with being able to adapt to change, have choice and a sense of personal freedom. Social connectedness was seen as the keystone to support healthy behaviors. |
| Howell, B. M. 2020. **USA** [113] | Cross-sectional survey, participant observation and individual interviews | n=82, 65+, mean age 74 | To examine the relationship between the sociocultural factors that shape diet, physical activity, and nutritional status outcomes among seniors. | T-tests indicate that diet and physical activity practices in this sample do not meet national recommendations and that diet differs adversely from national reference samples. Family influences increased fruit consumption, while participation in cultural and social events increased intake of fats and sweets. |
| Nogueira, D., et al. 2019. **Portugal** [114] | Three phase evaluation study | n=105 patients (with dysarthria), 18-102 years, mean age 64; and n=103 control (without), 18-87, mean age 50.6 | To produce a European Portuguese version of the Quality of Life Questionnaire for the Dysarthric Speaker (QoL-DyS). | The QoLDyS correlated positively with the QAD, PEAT-10, and EQ5D. Cronbach’s α was 0.973, and it remained excellent when any item was deleted. |
