# Supplementary material for: Eating Alone or Together among Community-Living Older People—A Scoping Review
Source: Int J Environ Res Public Health. 2021 Mar 27;18(7):3495. doi: 10.3390/ijerph18073495 (PMC8036467; doi:10.3390/ijerph18073495)
Supplement: Supplementary file 1 [file ijerph-18-03495-s001.zip › Appendix D.docx]

APPENDIX 4

Table 6 Geographical spread of included articles (n=72), also distributed in respective categories. The geographical origin of articles was divided in accordance with the regional classification of the United Nations [115]

| Geographic regions Total Central topic One aspect Peripheral | | | | |
| --- | --- | --- | --- | --- |
| Northern Africa | 0 (0%) |  |  |  |
| Sub-Saharan Africa | 2 (2%) |  | 2 |  |
| Northern America | 39 (40%) | 7 | 26 | 6 |
| Caribbean | 0 (0%) |  |  |  |
| Central America | 1 (1%) |  | 1 |  |
| Latin America | 0 (0%) |  |  |  |
| Central Asia | 0 (0%) |  |  |  |
| Eastern Asia | 16 (16%) | 13 | 1 | 2 |
| South-Eastern Asia | 0 (0%) |  |  |  |
| Southern Asia | 1 (1%) |  |  | 1 |
| Western Asia | 3 (3%) | 1 | 2 |  |
| Northern Europe | 23 (24%) | 8 | 12 | 3 |
| Eastern Europe | 0 (0%) |  |  |  |
| Southern Europe | 4 (4%) |  | 2 | 2 |
| Western Europe | 4 (4%) |  | 2 | 2 |
| Oceania | 5 (5%) | 1 | 2 | 2 |
|  | 98 (100%) | 30 | 50 | 18 |
